# Effect of Loureirin B on Crohn’s disease rat model induced by TNBS via IL-6/STAT3/NF-κB signaling pathway

**DOI:** 10.1186/s13020-019-0282-5

**Published:** 2020-01-06

**Authors:** Xueliang Sun, Ke Wen, Zhizhong Xu, Zongqi He, Bensheng Wu, Xiao Yang, Xiaopeng Wang

**Affiliations:** Department of Colorectal Surgery, Suzhou TCM Hospital Affiliated to Nanjing University of Chinese Medicine, Suzhou, 215000 Jiangsu China

**Keywords:** CD, LB, Inflammation, Fibrosis, IL-6/STAT3/NF-κB

## Abstract

**Background:**

Crohn’s disease (CD) is a chronic relapsing form of inflammatory bowel disease, seriously threatening human beings health. However, the pathogenesis of CD is still unclear and there is no specific effective drug for treatment of CD. Resina Donis (RD) obtained from *Dracaena cochinchinensis (Lour.) S. C. Chen* (Liliaceae), has been used for the treatment of CD clinically. Loureirin B (LB) is one of the most important chemical compositions and physiologically active ingredients of resina draconis. It has the molecular structure propan-1-one, 1-(4-hydroxyphenyl)-3-(2,4,6-trimethoxyphenyl)-1-(4-hydroxyphenyl)-3-(2,4,6-trimethoxyphenyl) propan-1-one. The aim of this study was to investigate the effect of LB on CD and explore the underlying mechanisms.

**Methods and results:**

In this study, the result demonstrated that LB prolonged the survival time of 2,4,6-trinitrobenzene sulfonic acid (TNBS)-induced rats and alleviated colonic damage in a dose dependent manner. Besides, LB remarkably ameliorated TNBS-induced inflammatory response via regulation of cytokines in the colonic tissues. Moreover, LB could reverse the established fibrosis and impede the accumulation infiltration, and improve the apoptosis induced by TNBS in a dose dependent manner. Further, LB dramatically suppressed TNBS-induced the activation of IL-6/STAT3/NF-κB signaling pathway.

**Conclusions:**

These findings suggested that LB could be beneficial regarding ameliorating TNBS-induced CD, which may represent a novel approach to treat CD and provide an alternative choice for disorders associated with CD.

## Background

CD is a chronic relapsing form of inflammatory bowel disease, typically characterized with transmural inflammation, lymphangiectasia, and lymphatic and fibrous tissue hyperplasia [1, 2]. CD can be involved in all segments of the digestive tract, and bring about perforation and abdominal adenoma and other complications, which seriously affects the life quality of patients [3]. Thus, CD is a difficult disease urgently needed to be solved. CD occurs mostly in developed countries such as Europe and America, the incidence of CD is 43.6 per 100,000 in Europeans and Americans, and 5.6 per 10 million in Asians [4]. In recent years, with the changes of living conditions and the continuous improvement of clinical diagnosis level, the incidence of CD in China has been increasing year by year [5, 6]. Compared with the clinical diagnosis of CD, the research progress of the treatment for CD is relatively slow. In addition, the pathogenesis of CD is still unclear, and CD is thought to be the results of heredity, immune disorders, intestinal barrier dysfunction and intestinal microbial interaction [7]. It is precisely because the current research can not reveal the exact pathogenesis of CD, so there are no specific effective treatment methods or drugs for CD [8].

CD is mainly characterized by nonspecific inflammation of the digestive tract, which occurs in the individual intestinal tract with genetic susceptibility and does not depend on specific pathogens [9]. Studies have shown that lamina propria T cells play a key role in the induction and persistence of intestinal inflammation. Interaction between antigen presenting cells in intestinal mucosal immune system and local bacterial flora leads to the uncontrolled activation of mucosal CD4^+^ T cells, and sustained release of inflammatory factors such as tumor necrosis factor-α (TNF-α), interleukin-6 (IL-6), IL-12, IL-23, IL-27 and IL-17, which are important factors resulting in intestinal injury in CD patients [10, 11]. IL-6 can be produced by multiple cells and has pleiotropic effects on different organ systems. In the pathological state of CD, CD4^+^ T cells exhibit apoptosis resistance, and the accumulation of CD4^+^ T cells in inflammatory sites mainly depends on anti-apoptotic IL-6 trans-membrane signaling pathway [12]. IL-6 trans-membrane signaling pathway can induce the activation of STAT3, and STAT3 itself can further induce the expression of anti-apoptotic factors Bcl-2 and Bax-xl, causing T cells resistance to apoptosis [13, 14]. Besides, T cell apoptosis resistance and aggregation eventually lead to a vicious cycle of chronic inflammation, which can be effectively blocked by anti-IL-6 receptor antibody, indicating that IL-6/STAT3 signaling pathway plays a central role in apoptosis resistance in the pathological state of CD [15]. In addition, recent studies have found that IL-6 signaling pathway plays an important role in the occurrence and differentiation of Th17, suggesting that IL-6/STAT3 trans-membrane signaling pathway is essential for T cells apoptosis resistance and type Th17 immune reaction [16, 17]. Further, in inflammatory cells, the inappropriate activation of nuclear factor-кB (NF-кB) is the key transcription factor that regulates the expression of the inflammatory mediators, which is related to the occurrence and development of CD inflammation [18].

RD, obtained from *Dracaena cochinchinensis (Lour.) S. C. Chen* (Liliaceae), is a Chinese herb with the positive activities of promoting blood circulation for removing blood stasis, regenerating tissue to heal wound, relieving pain and eliminating swelling, which has been commonly used for the treatment of coronary heart disease, angina, and acute myocardial infarction [19, 20]. Additionally, the ethyl acetate of RD can promote inflammatory response induced by LPS through inhibiting ROS production in vascular smooth muscle cells and macrophages [21]. Moreover, the clinical effect of RD on CD is satisfactory in China [22]. LB is one of the most important chemical compositions and physiologically active ingredients of resina draconis. It has the molecular structure propan-1-one, 1-(4-hydroxyphenyl)-3-(2,4,6-trimethoxyphenyl)-1-(4-hydroxyphenyl)-3-(2,4,6-trimethoxyphenyl) propan-1-one. The previous study reported that LB could inhibit the hepatic stellate cell proliferation by regulating miR-148-3p via Wnt/β-catenin signaling pathway [23]. LB, an essential component of Sanguis Draxonis, inhibits Kv1.3 channel and suppresses cytokine release from Jurkat T cells [24]. LB inhibited fibroblast proliferation and extracellular matrix deposition in hypertrophic scar via TGF-β/Smad pathway [25]. However, the role of LB on CD and the underlying mechanisms remain unknown. Therefore, in the current study, we investigated the effect of LB on CD rat model induced by TNBS and explored the possible mechanisms.

## Methods

### Experimental materials

Sixty Sprague–Dawley (SD) rats (equal ratio of male and female), weighing 250 ± 20 g, were obtained from Experimental Animal Center of Nanjing University (Nanjing, China). Sulphasalazine (SASP) (250 mg) was purchased from the National Institutes for Food and Drug Control (Beijing, China). Trinitro-benzene-sulfonic acid (TNBS) was purchased from Sigma Chemical (St. Louis, MO, USA).

### Preparation of LB

LB in the present study was followed as previous recommendations [26]. LB was obtained from the National Institute for the Control of Pharmaceutical and Biological Products of China and reconstituted in DMSO at a final stock concentration of 25 mg/mL.

### Establishment of the CD rat model

The CD rat model was induced using TNBS as described as previous [27]. In brief, SD rats were fasted overnight and lightly anesthetized with ether. Then, TNBS (5 mg), dissolved in 0.2 mL of 50% ethanol, was injected into the descending colon, and the rats from the control group were only treated with 0.2 mL of 50% ethanol following the same method once a week for a total of four treatments.

### Experimental animals grouping and treatments

CD rats were randomly divided into six groups (n = 10), including the control group, the model group, the SASP group, and the LB groups (25, 50 and 100 mg/kg). In the control group, rats were only administered with normal saline via oral gavage every day. The rats from the model group were treated with TNBS once a week for a total of four treatments and administrated with normal saline via oral gavage every day. The rats of the SASP group were treated with prepared SASP suspension liquid (0.1 g/mL) daily via oral gavage based on the model group, SASP was an effective drug for CD [28, 29]. The LB group rats were administered with 25, 50 and 100 mg/kg LB every day for a total of 28 d via oral gavage based on the model group.

### Assessment of colonic damage

The disease activity index (DAI) of all the rats from different groups was evaluated daily according to criteria [30, 31] (Table 1). Every group rats were checked the weight and euthanized at day 28. Then the distal colon was carefully excised and the colon was weighed and measured length.

**Table 1:** Criteria for assessment of colonic macroscopic and microscopic damage

Score	Body weight loss	Stool character	Fecal occult blood
0	0	Normal formed	Negative
1	= 1 to 5%		
2	= 5 to 10%	Loose stool	Positive
3	= 10 to 20%		
4	> 20%	Diarrhea	Gross bleeding

### Hematoxylin and eosin (H&E) staining

Colonic segments were excised and washed with phosphate buffered saline (PBS). Then the sections were dehydrated by alcohol, cleared in xylene and embedded in paraffin. The colonic sections of 5 μm thickness were transversely cut and stained with H&E for visual analysis.

### Masson staining assay

Isolated colonic tissues were fixed in 4% neutral formalin and embedded in paraffin. Then the sections (5 μm) were stained with Masson trichrome solutions. Images were obtained using a light microscope.

### TUNEL assay

Apoptosis was analyzed using a terminal deoxynucleotidyl transferase-mediated dUTP-biotin nick end labeling (TUNEL) assay kit following the manufacturer’s instructions. In brief, the sections (5 μm) of isolated colonic tissues were incubated with fluorescein isothiocyanate (FITC)-labeled dUTP and terminal deoxynucleotidyl transferase for 1 h at 37 °C. Nuclei counterstaining was performed using DAPI, and examined under a fluorescence microscope (Olympus, Tokyo, Japan).

### Immunohistochemical analysis

Isolated colonic tissues from different groups were immunohistochemically stained for Slug (ab27568; Abcam; 1:1000 dilution), Snail (ab53519; Abcam; 1:500 dilution), N-cadherin (ab18203; Abcam; 1:300 dilution), E-cadherin (ab1416; Abcam; 1:100 dilution), Vimentin (ab8978; Abcam; 1:200 dilution), Bax (ab32503; Abcam; 1:250 dilution), Bcl-2 (ab32124; Abcam; 1:500 dilution), Caspase-3 (ab13847; Abcam; 1:500 dilution), Caspase-9 (ab32539; Abcam; 1:250 dilution). Briefly, isolated colonic tissues were fixed in 4% neutral formalin for 24 h, embedded in paraffin and were serially sectioned at 5 µm. Sections were deparaffinized and rehydrated, then submerged in hydrogen peroxide to quench peroxidase activity following incubated with 1% BSA to block non-specific binding sites. After incubation with indicated primary antibodies at 4 °C for 12 h, secondary antibodies IgG (ab6721; Abcam; 1:1000 dilution) were applied to slides for 1 h at room temperature. For immunohistochemical analysis, the slice was colored by diaminobenzidine kit (DAB; Beyotime) followed by counterstaining with hematoxylin. All the sections were visualized using diaminobenzidine (DAB, Beyotime) under a light microscope (Nikon 80i).

### Enzyme-linked immunosorbent assay (ELISA)

The concentrations of cytokines in isolated colonic tissues were determined by enzyme-linked immunosorbent assay (ELISA) for rat IL-1, IL-1β, IL-6, IL-10, TNF-α, TGF-β, INOS, MPO, SIgA and MDA (eBioscience, San Diego, CA) following the manufacturer’s instructions.

### Quantitative real-time PCR assay (qRT-PCR)

Total RNA of isolated colonic tissues was extracted with Trizol reagent (Takara, China) and was reverse-transcribed into cDNA using a PrimeScript RT reagent kit (Takara, China) according to the manufacturer’s instructions. Quantitative PCR (ABI 7500, USA) was performed using SYBR green kit (Takara, China) to detect the relative mRNA expression levels of IL-6, p-STAT3, STAT3, p-NF-κB (p65) and NF-κB (p65). GAPDH was normalized to an endogenous reference. The relative gene expression level was calculated by using the comparative Ct method, and those relative to the calibrator were given by the formula 2^−ΔΔCt^. The PCR primers for IL-6 were 5′-GAGAAAAGAGTTGTGCAATGGC-3′ (forward) and 5′-ACTAGGTTTGCCGAGTAGAC C-3′ (reverse), STAT3 were 5′-GAGGAGGCATTCGGAAAGTATT-3′ (forward) and 5′-CAGGT CGTTGGTGTCACACA-3′ (reverse), NF-κB (p65) were 5′-CACCAAAGACCCACCTCACC-3′ (forward) and 5′-CCGCATTCAAGTATAG TCCC-3′ (reverse), GAPDH were 5′-CGCTAACATC AAATGGGGTG-3′ (forward) and 5′-ACAACCTGGTCCTCAGTGTA-3′ (reverse).

### Western blotting assay

The total protein from the isolated colonic tissues was extracted using RIPA lysis buffer (Pierce; Rockford, IL, USA) according to the manufacturer’s instructions. Protein concentrations were measured with BCA protein assay kit. Equal amounts of protein samples (50 μg) were separated on 12% sodium dodecyl sulfate-polyacrylamide gel electrophoresis (SDS-PAGE) and transferred to a polyvinylidene difluoride membrane (PVDF) (Millipore; Billerica, MA, USA). Then the PVDF membrane was blocked with 5% bovine serum album (Amresco, Solon, OH, USA) for 1 h and incubated with primary antibodies including Slug (cat. no. ab27568; Abcam), Snail (cat. no. ab53519; Abcam), N-cadherin (cat. no. ab18203; Abcam), E-cadherin (cat. no. ab1416; Abcam), Vimentin (cat. no. ab8978; Abcam), Bax (cat. no. ab32503; Abcam), Bcl-2 (cat. no. ab32124; Abcam), Caspase-3 (cat. no. ab13585; Abcam), Caspase-9 (cat. no. ab32539; Abcam), IL-6 (cat. no. ab6672; Abcam), p65 (cat. no. ab16502; Abcam), IKB-α (cat. no. ab32518; Abcam) and GAPDH (cat. no. ab9485; Abcam) at 1:1000 dilution at 4 °C overnight. After washing with PBS three times, the membranes were further incubated with horseradish peroxidase-conjugated secondary antibody IgG (cat. no. ab6721; 1:2000; Abcam). The blots were visualized using the ECL-plus detection system (GE Healthcare, Buckingham-shire, UK). The membrane was exposed to X-ray film and then developed. The signal intensity was quantified using Gray-scale analysis software (Image Tool 3.00).

### Statistical analysis

Graphpad Prism 5.0 software was performed to analyze all data. The data were represented as mean ± SD from at least three independent assays. One-way analysis of variance followed by Tukey was applied to compare difference between multiple groups. Additionally, non-parametric tests were applied for data with non-fulfilled distribution assumptions due to some normalized data. A p-value < 0.05 was considered statistically significant.

## Results

### Effect of LB on the survival rate and body weight in rats induced with TNBS

**Fig. 1:**
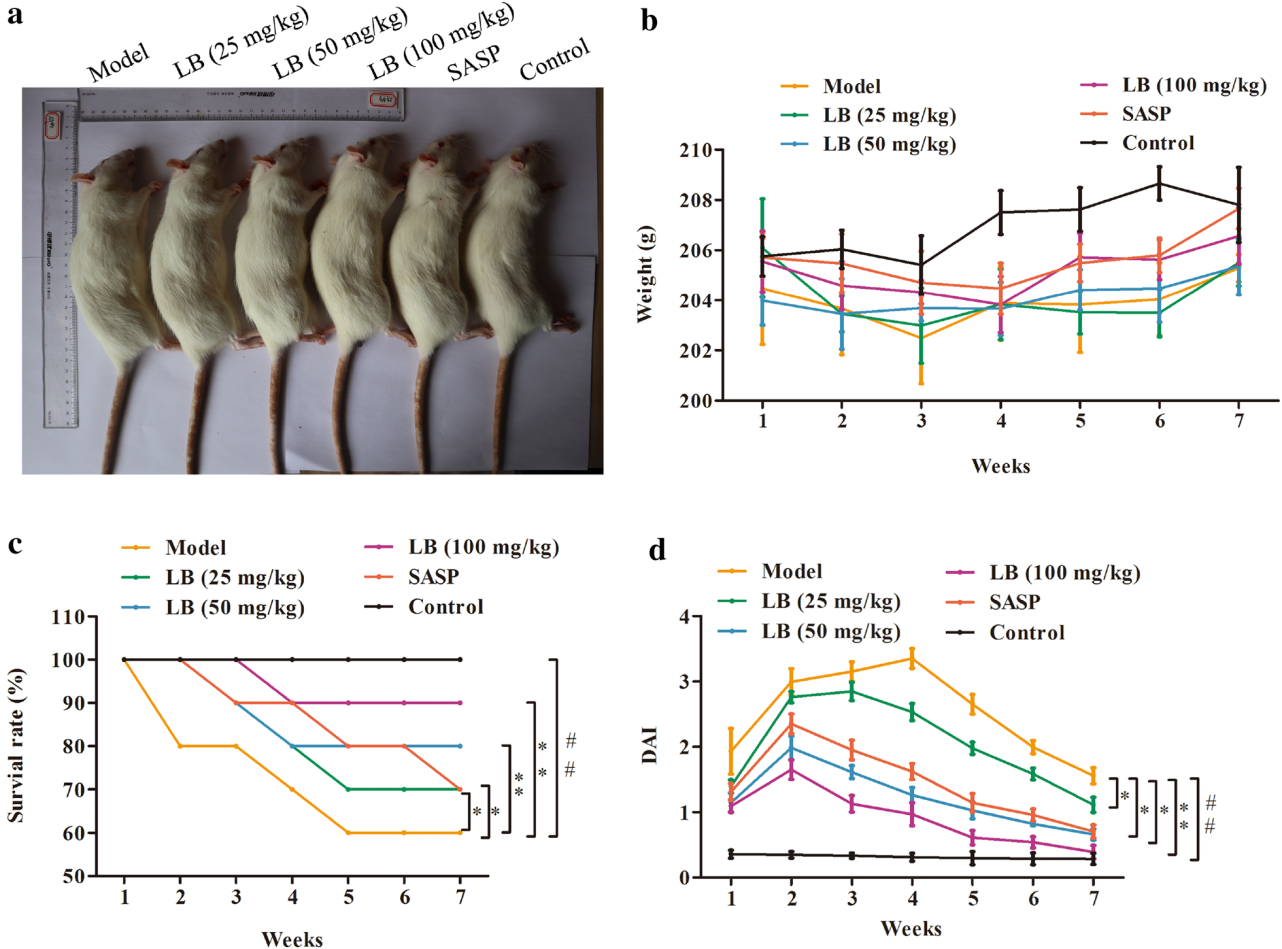
Effect of LB on the survival rate and body weight in rats induced with TNBS. **a**, **b** The body weight loss of CD rats treated with LB was evaluated. F = 9.594, df = 41. **c** The survival time of CD rats treated with LB was measured. F = 5.604, df = 41. **d** The DAI score of rats from different groups was counted. F = 15.32, df = 41. The results were expressed as the mean ± SD of at least three experiments. ^##^*P* < 0.01 compared with the control group. *P < 0.05, **P < 0.01 compared with the model group

Firstly, to evaluate whether LB had a protective effect on the rats induced with TNBS, the survival rate and the body weight of TNBS-induced rats were measured. As shown in Fig. 1a, b, TNBS treatment could dramatically decrease the rat body weight, whereas treatment with LB appeared to increase the body weight in a dose dependent manner. Besides, the results demonstrated that the survival rate of rats from the TNBS was lower compared with that in the control group, while the survival time of rats was prolonged after treatment of LB in a dose dependent manner, and the effect of LB with high concentration was better than SASP treatment (Fig. 1c). Moreover, rats from different groups also had intense inflammatory response characterized by DAI. Saline did not protect against colonic injury. However, treatment with SASP significantly inhibited the colonic injury, whereas administration with LB could protect the rat colon against TNBS-induced injury (Fig. 1d).

### Effect of LB on the colonic length in rats induced with TNBS

**Fig. 2:**
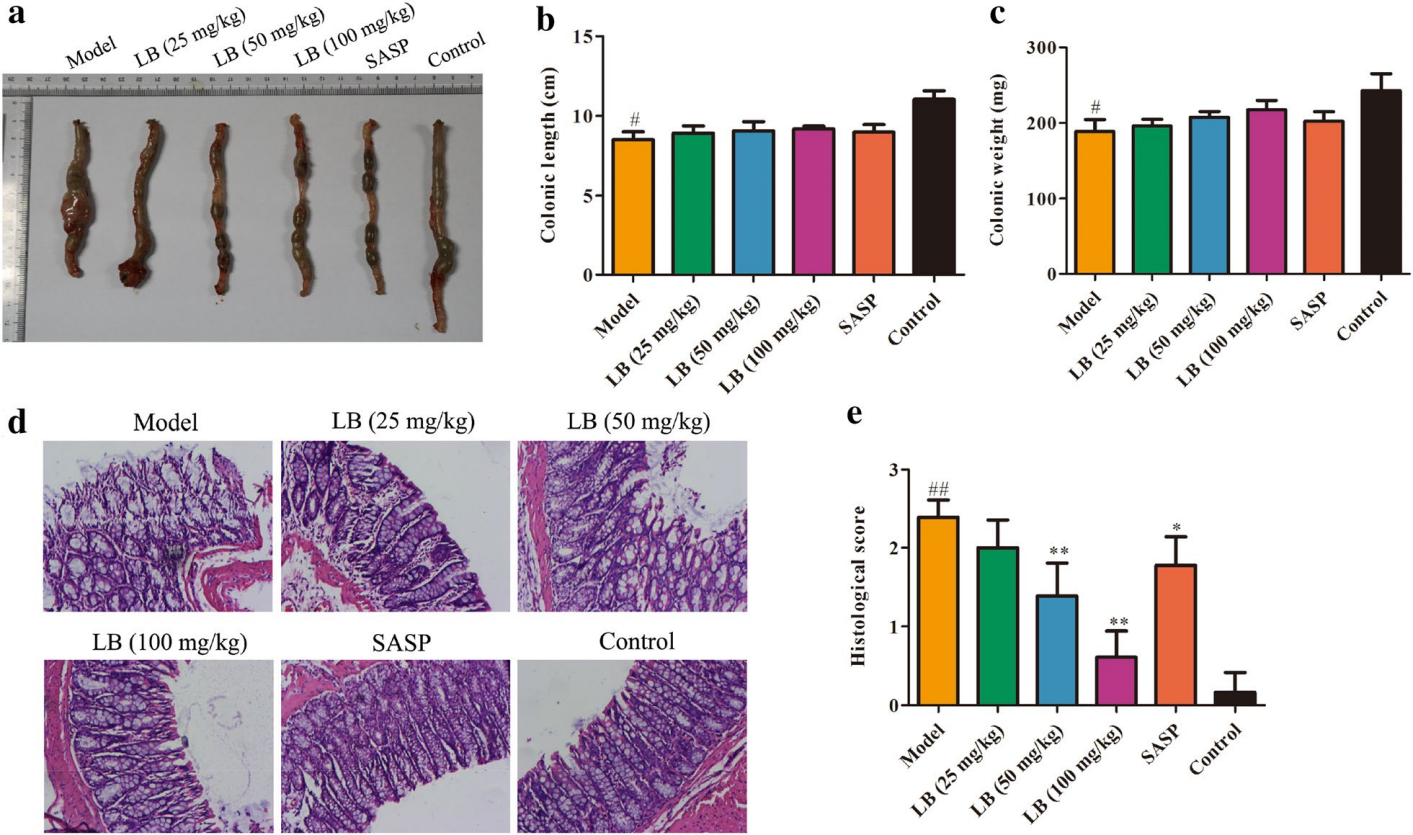
Effect of LB on the colonic length in rats induced with TNBS. **a**–**c** The colonic length (F = 10.79, df = 17) and weight (F = 5.472, df = 17) of CD rats treated with LB was measured. **d**, **e** The severity of colonic inflammation of CD rats treated with LB was evaluated by H&E staining. F = 59.95, df = 53. The results were expressed as the mean ± SD of at least three experiments. ^#^*P* < 0.05, ^##^*P* < 0.01 compared with the control group. *P < 0.05, **P < 0.01 compared with the model group

Colon shortening is an indirect marker of inflammation [32]. Compared with the control group, the colonic length in model group was significantly decreased, which demonstrated that CD rats had seriously colon injury. After treatment with different concentrations of LB, no obvious change was viewed in the colonic length and weight (Fig. 2a–c). Moreover, the severity of colonic inflammation was evaluated by H & E staining assay. As shown in Fig. 2d, e, the architecture of colonic tissues from the control group was normal and was devoid of any inflammatory infiltration necrosis and edema. Rats induced with TNBS caused a significant elevation in inflammatory response characterized by thickening of the mucosa, inflammatory cell infiltration with necrotic foci, extensive destruction of mucosal epithelium, loss of mucus-secreting cells, submucosal edema, necrosis and ulcer on the mucosal surface. The colonic tissues from SASP group exhibited a downward trend in the inflammatory infiltration, and rats treated with LB showed remarkable inhibitory effect on TNBS-induced colonic histological damage in a dose dependent manner.

### Effect of LB on the production of inflammatory cytokines in rats induced with TNBS

**Fig. 3:**
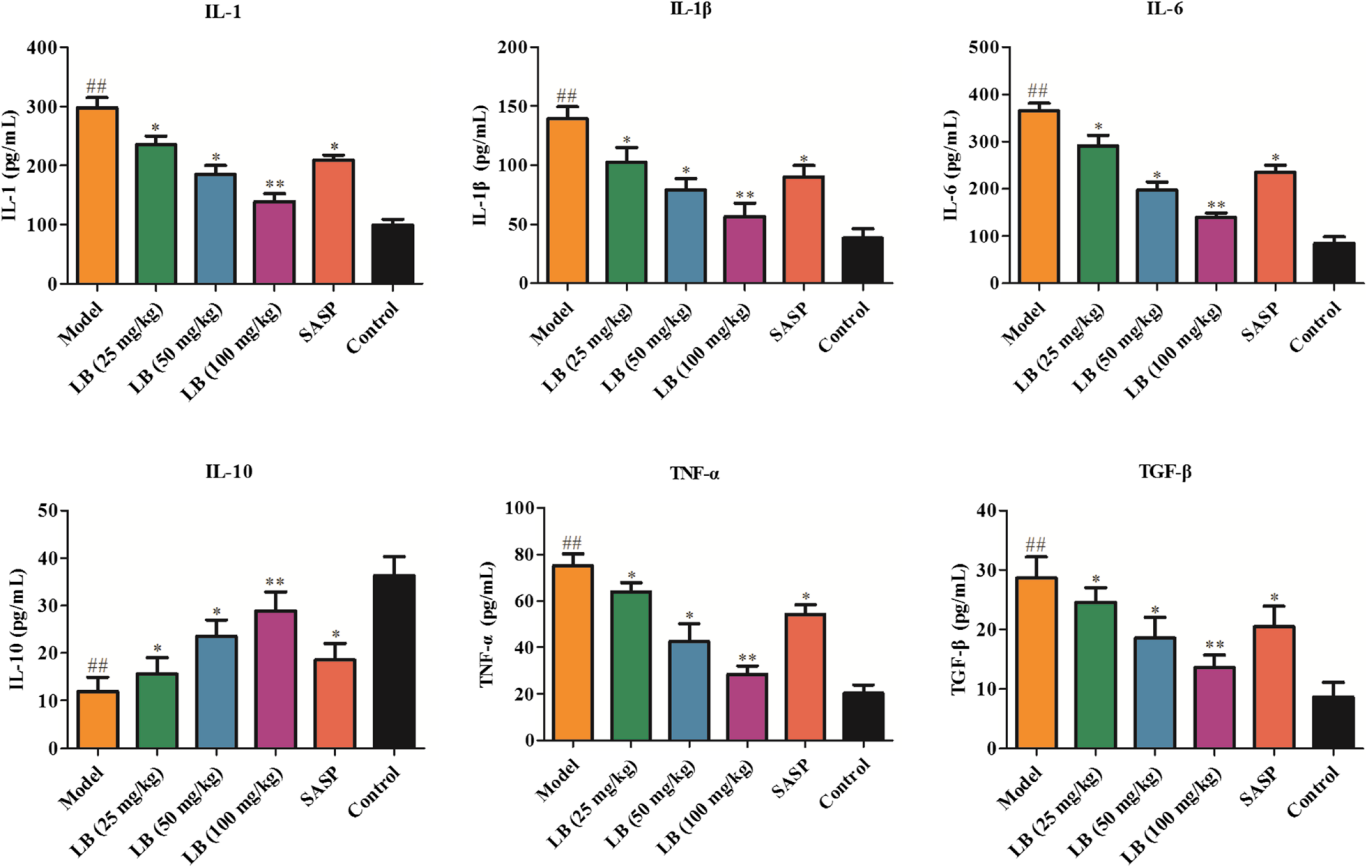
Effect of LB on the production of inflammatory cytokines in rats induced with TNBS. ELISA assay was performed to detect the production of cytokines, including IL-1 (F = 78.32, df = 17), IL-1β (F = 35.24, df = 17), IL-6 (F = 119.5, df = 17), TNF-α (F = 57.35, df = 17), IL-10 (F = 18.79, df = 17) and TGF-β (F = 17.67, df = 17) in colonic tissues of CD rats from different groups. The results were expressed as the mean ± SD of at least three experiments. ^##^*P* < 0.01 compared with the control group. *P < 0.05, **P < 0.01 compared with the model group

In order to investigate the effect of LB on the production of inflammatory cytokines in rats induced with TNBS, the colonic tissues were collected. As described in Fig. 3, TNBS could significantly elevate the production of IL-1, IL-1β, IL-6, TNF-α and TGF-β, and decrease the secretion of IL-10. However, LB treatment could obviously reverse these phenomenons, which was that the administration of LB in rat led to a decrease of the production of IL-1, IL-1β, IL-6, TNF-α, IL-6, TGF-β and an increase of the production of IL-10. These results indicated that LB had a positive role in the modulation of cytokine production in CD rats induced with TNBS.

### Effect of LB on the expression of INOS, MPO, SIgA and MDA in rats induced with TNBS

**Fig. 4:**
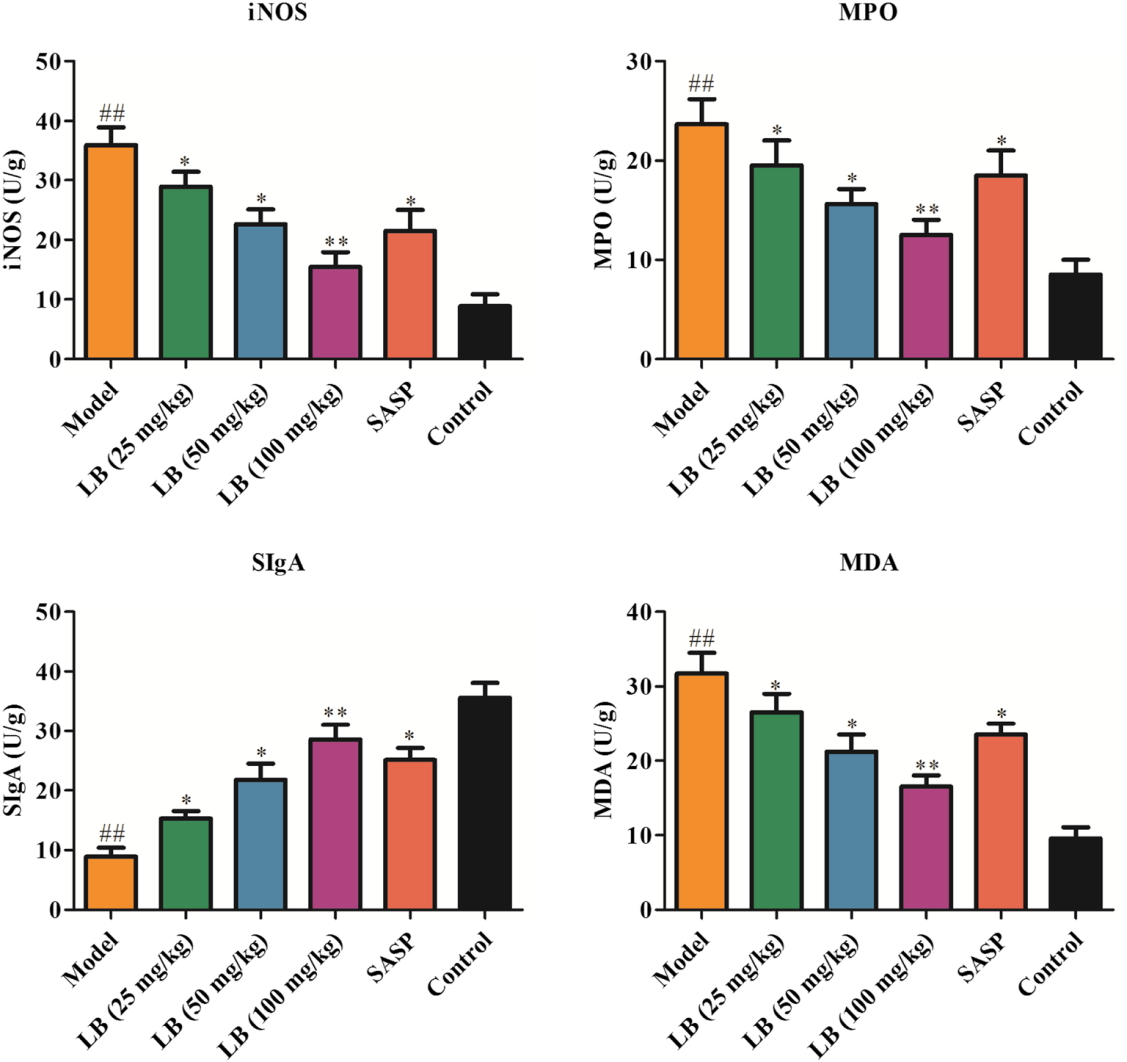
Effect of LB on the expression of INOS, MPO, SIgA and MDA in rats induced with TNBS. ELISA assay was performed to detect the production of INOS (F = 36.97, df = 17), MPO (F = 20.34, df = 17), SIgA (F = 58.30, df = 17) and MDA (F = 42.37, df = 17) in colonic tissues of CD rats from different groups. The results were expressed as the mean ± SD of at least three experiments. ^##^*P* < 0.01 compared with the control group. *P < 0.05, **P < 0.01 compared with the model group

To further explore the role of LB in the expression of INOS, MPO, SIgA and MDA related to the infiltration of neutrophils in inflammatory tissues [33], we performed ELISA assay to detect the expression of INOS, MPO, SIgA and MDA in colonic tissues. As shown in Fig. 4, the expression of INOS, MPO and MDA was up-regulated and the expression of SIgA was down-regulated in TNBS-induced group compared to that in the control group, while treatment with LB led to a decrease in the expression of INOS, MPO and MDA and an increase of SIgA in colonic tissues.

### Effect of LB on epithelial-mesenchymal transition (EMT) in rats induced with TNBS

**Fig. 5:**
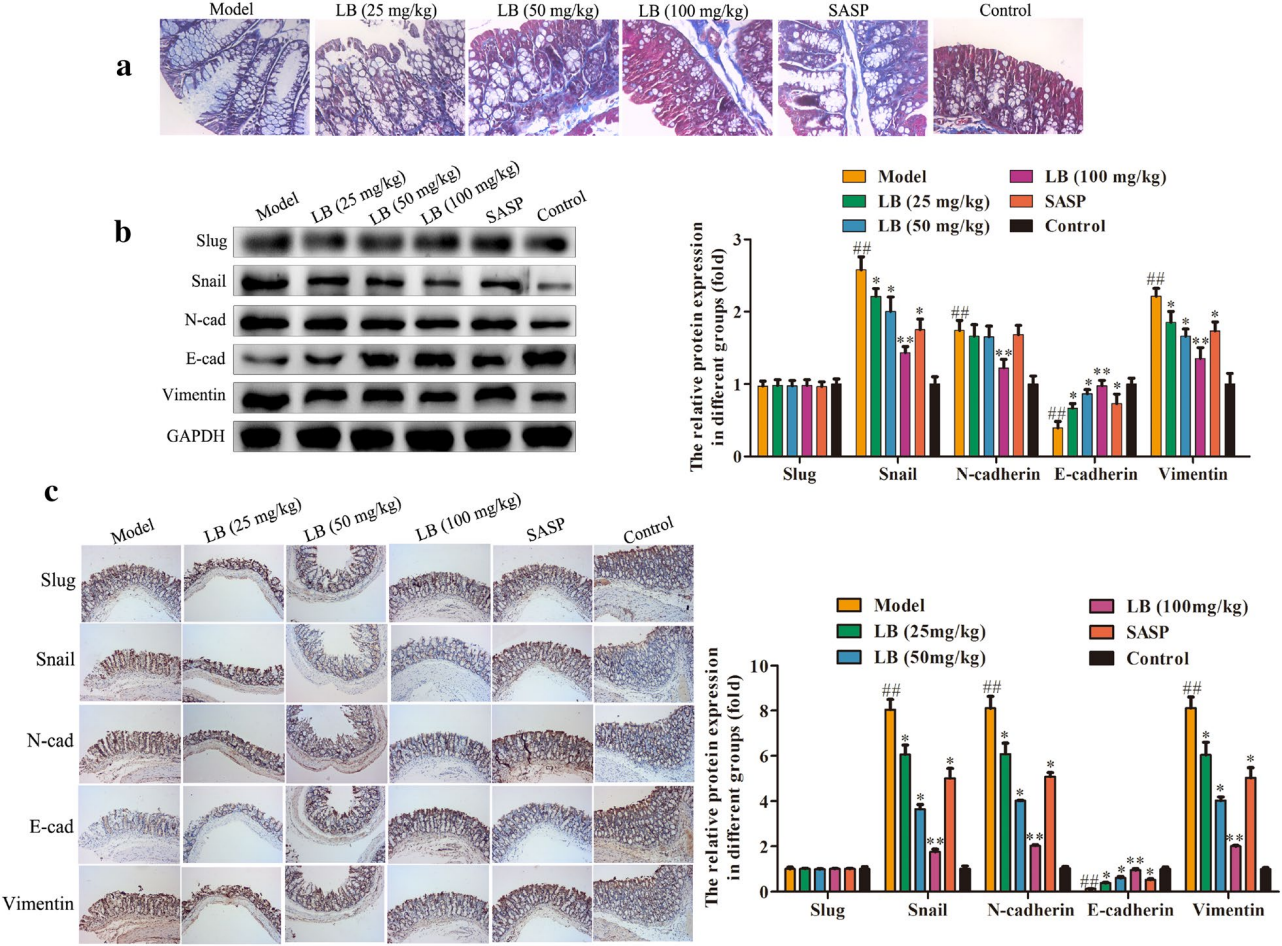
Effect of LB on epithelial-mesenchymal transition (EMT) in rats induced with TNBS. **a** Masson’s trichrome staining assay was performed to investigate the fibrotic changes histopathologically of colonic tissues of CD rats from different groups. **b** Western blotting assay was carried out to examine the expression levels of EMT-related proteins, including Slug, Snail, N-cadherin, E-cadherin and Vimentin of colonic tissues of CD rats from different groups. The band intensity was quantified by Image J software. F = 0.7712, df = 29. **c** Immunohistochemistry assay was performed to evaluate the expression levels of EMT-related proteins in colonic tissues of CD rats from different groups. F = 1.989, df = 29. The results were expressed as the mean ± SD of at least three experiments. ^##^*P* < 0.01 compared with the control group. *P < 0.05, **P < 0.01 compared with the model group

Intestinal fibrosis is thought to be a common phenomenon in inflammatory bowel disease, and often causes serious complications in CD, eventually leading to intestinal stenosis [34]. Masson’s trichrome staining assay was performed to investigate the fibrotic changes histopathologically. The results of Fig. 5a demonstrated that TNBS could promote the inflammatory infiltration and fibrotic tissue accumulation, while LB treatment could reverse the established fibrosis and impeded the accumulation infiltration. Additionally, EMT has been found to be an important pathway in the progress of intestinal fibrosis recently. Western blotting assay was carried out to examine the expression levels of EMT-related proteins, including Slug, Snai, N-cadherin, E-cadherin and Vimentin. As shown in Fig. 5b, the expression levels of Snail, N-cadherin and Vimentin were obviously over-expressed, and the expression levels of E-cadherin was notably low-expressed in rat induced with TNBS. After treatment with LB, the expression levels of Snail, N-cadherin and Vimentin were significantly down-regulated and the expression level of E-cadherin was remarkably up-regulated in a dose depend manner. However, there were no significant changes in the expression of Slug. Besides, immunohistochemistry assay was performed to evaluate the expression levels of these proteins. The trend of the results was consistent with the trend of western blotting assay (Fig. 5c).

### Effect of LB on apoptosis in rats induced with TNBS

**Fig. 6:**
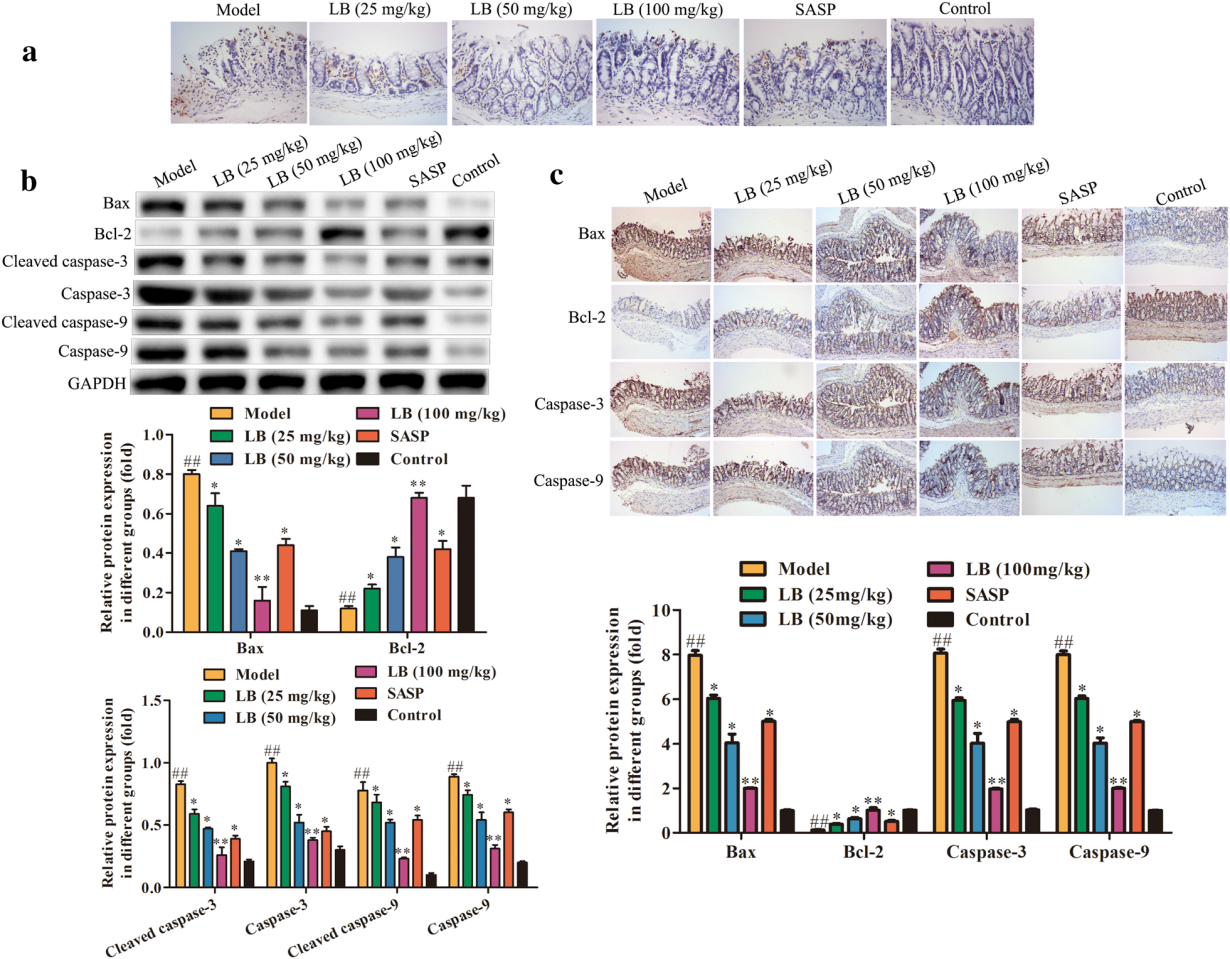
Effect of LB on apoptosis in rats induced with TNBS. **a** TUNEL assay was performed to evaluate the apoptotic degree of colonic tissues of CD rats from different groups. **b** Western blotting assay was adapted to examine the expression levels of proteins related to apoptosis of colonic tissues of CD rats from different groups. The band intensity was quantified by Image J software. F = 4.658, df = 35. **c** Immunohistochemistry assay was performed to evaluate the expression levels of proteins related to apoptosis in colonic tissues of CD rats from different groups. F = 2.596, df = 23. The results were expressed as the mean ± SD of at least three experiments. ^##^*P* < 0.01 compared with the control group. *P < 0.05, **P < 0.01 compared with the model group

Apoptosis is a programmed cell death occurring in various inflammatory diseases including CD [35]. Firstly, TUNEL assay was performed to evaluate the apoptotic degree of colonic tissues. As shown in Fig. 6a, TNBS could obviously promote the colonic tissue apoptosis, while treatment with LB had better improving effect on the apoptosis induced by TNBS in a dose dependent manner. Further, western blotting and immunohistochemistry assays were adopted to examine the expression levels of proteins related to apoptosis. The results of Fig. 6b, c demonstrated that TNBS could promote the expression levels of Bax, Cleaved-caspase-3 and Cleaved-caspase-9, and suppress the expression of Bcl-2. Interestingly, LB treatment could reverse the effect of TNBS.

### Effect of LB on IL-6/STAT3/NF-κB signaling pathway in rats induced with TNBS

**Fig. 7:**
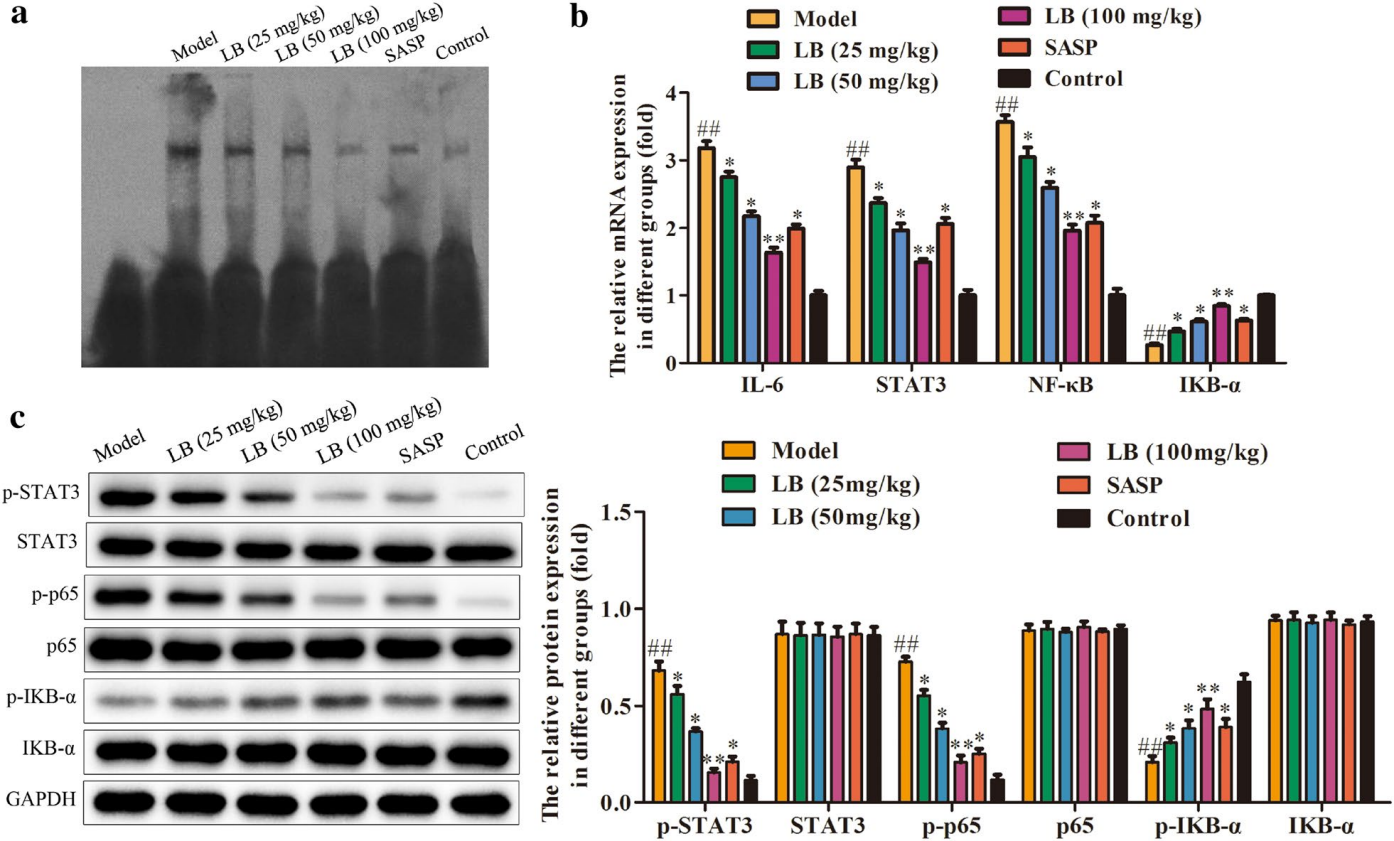
Effect of LB on IL-6/STAT3/NF-κB signaling pathway in rats induced with TNBS. **a** The functional activity of NF-κB was accessed by EMSA. **b**, **c** qRT-PCR and western blot assays were performed to evaluate the mRNA and protein expression of IL-6/STAT3/NF-κB signaling pathway of colonic tissues of CD rats from different groups. The band intensity was quantified by Image J software. The results were expressed as the mean ± SD of at least three experiments. ^##^*P* < 0.01 compared with the control group. *P < 0.05, **P < 0.01 compared with the model group

IL-6/STAT3/NF-κB signaling pathway plays an important role in the development and progression of CD [36]. The functional activity of NF-κB was accessed by electrophoretic mobility shift assay (EMSA) (Fig. 7a). In this study, we further investigated the effect of LB on the expression of IL-6/STAT3/NF-κB in CD rats by using qRT-PCR and western blot assays. As shown in Fig. 7b, c, the abundances of IL-6, p-STAT3, p-NF-κB (p65) were greatly increased and p-IKB-α was decreased in model group compared with control group. However, it was observed that SASP treatment lowered the expression of IL-6, p-STAT3, p-NF-κB (p65) and raised the expression of p-IKB-α as compared with the model group. Moreover, LB treatment had better inhibitory effects on the expression of IL-6/STAT3/NF-κB signaling pathway.

## Discussion

CD is a transmural, segmental, asymmetric inflammatory bowel disease that affects any part of the digestive tract [1, 2]. This disease is more common in European and American countries, but recently the incidence of CD is increasing obviously in China, and the incidence of CD between 2004 and 2008 is 8.5 times higher than that between 1989 and 1993 in China [37, 38]. In addition, the etiology and pathogenesis of CD are not yet fully understood. At present, it is believed that CD may be caused by mucosal damage caused by intestinal immune hyperactivity due to the infection, diet and other environmental factors. Immune abnormalities have been recognized to play a very important role in the pathogenesis of CD, including inflammatory transmitters, cytokines and immune regulation [39–41].

Nowadays, clinical treatment of CD is mainly based on drugs according to the severity of CD, and commonly used drugs are aminosalicylic acid, glucocorticoids, immunosuppressors and anti TNF-α biological agents [42]. Actually, 5-aminosalicylic acid (5-ASA) can not effectively alleviate CD, the curative effect is uncertain, and has many side effects [43]. Clinical studies have shown that the abuse of glucocorticoids and immunosuppressors not only can not control the degree of CD, but also cause seriously drug-related complications, leading to serious damage to the body [44]. As the most advanced medical method for CD at present, the treatment efficiency of the combination of anti-tumor necrosis factor and mercaptopurine is only about 50% [45, 46]. Therefore, most of the drugs and treatments currently used in the treatment of CD clinically have poor efficacy and side effects.

Over the years, Chinese medicine has accumulated a wealth of experience in the treatment of CD, a large number of literature reports and modern research have confirmed that Chinese medicine in the treatment of CD has a significant effect [47–49]. LB as a Chinese herb has been commonly used for the treatment of coronary heart disease, angina, and acute myocardial infarction, and the ethyl acetate of LB can promote inflammatory response induced with LPS through inhibiting ROS production in vascular smooth muscle cells and macrophages [19–22]. However, the role of LB on CD and the underlying mechanisms remain unknown. In the present study, we found that LB could prolong the survival time of CD rats, ameliorate TNBS-induced weight loss, colonic length and inflammatory infiltration.

Recent studies have shown that CD is a typical type Th I reaction. In addition to immune cells, non-immune cells of intestinal mucosa, such as epithelial cells, vascular endothelial cells and interstitial cells, also participate in the immune response and inflammatory process of CD through releasing various cytokines and inflammatory mediators, which can lead to the occurrence and development of CD. Further, regulating the expression of some cytokines can treat CD [50, 51]. For example, high level of IL-6 is associated with active CD [52]. Nunberg et al. unveiled impaired IL-10 receptor-mediated suppression in monocyte from patients with Crohn’s disease [53]. In this study, we found that the administration of LB in rats induced with TNBS led to a significant decrease of the production of IL-1, IL-1β, IL-6, TGF-β and TNF-α, and an obvious increase of secretion of IL-10. Thereafter, we further measured the concentrations of some cytokines related to inflammation in CD, including INOS, MPO, SIgA and MDA. Previous literatures illuminated that the positive correlations of INOS, MPO and MDA with the severity of CD [54–56]. However, SIgA was lowly expressed in CD patients and SIgA might be involved in CD pathogenesis [57]. Our findings expounded that LB treatment ameliorated the expression of INOS, MPO and MDA in TNBS-induced rats, while the activity of SIgA was significantly inhibited by LB. These results indicated that LB had a positive role in the modulation of cytokine production in the setting of CD.

Chronic inflammation and injury of the intestine can lead to serious complications such as intestinal fibrosis and intestinal obstruction. Therefore, the research on the pathogenesis of CD intestinal fibrosis has become a popular direction [58]. Under normal physiological conditions, fibroblasts aggregate in damaged tissues to promote the repair of damaged tissues. The ability of fibroblasts in the intestinal mucosa to aggregate into damaged tissues in patients with CD is weakened and the wound healing function is disordered compared with normal people. When the intestine is damaged, the epithelial cells of the intestinal mucosa can migrate under the induction of many growth factors. When the fibroblasts in the intestinal tract can not repair the defect of mucosal tissues, intestinal epithelial cells can be transformed into interstitial cells, and migrate to the damaged area, so that the defective intestinal barrier can be repaired [59]. Intestinal epithelial cells may undergo EMT or apoptosis in the presence of persistent adverse environmental effects. In fact, the cytokine TGF-β, which plays an important role in the development of EMT, can promote the apoptosis of epithelial cells [60]. Exposure to TGF-β can induce apoptosis of a large number of epithelial cells. In addition, the weakening of adhesion between cells and basement membrane during EMT is also an important pathway of apoptosis [61]. Therefore, if EMT persists and fibroblast apoptosis is inhibited, it will lead to progressive development of intestinal fibrosis. In the present study, we found that LB treatment could reverse the established fibrosis and impeded the accumulation infiltration, and after treatment with LB, the expression levels of Snail, N-cadherin and Vimentin were significantly down-regulated and the expression level of E-cadherin was remarkably up-regulated in a dose depend manner. Further, treatment with LB had better improving effect on the apoptosis induced with TNBS in a dose dependent manner.

IL-6/STAT3 signaling pathway plays an important role in the occurrence and development of IBD [62]. The activation of IL-6/STAT3 signal transduction pathway mainly depends on the recognition and binding of IL-6 and sIL-6R on the target cell surface to form sIL-6R/IL-6 complex, which further activates gp130 on the surface of cell membrane. Gp130 is stimulated to form homologous dimers, which activates JAK associated with gp130 and receptor tyrosine kinase, and binds to STAT3 protein. This kinase cascade phosphorylates STAT3 and actives NF-κB signaling pathway, thereby regulating expression of inflammatory cytokines [63]. In this study, we found that LB could inhibit the activation of IL-6/STAT3/NF-κB signaling pathway.

## Conclusions

To conclude, our study suggested that LB could be beneficial regarding ameliorating the damage to colon length, suppressing inflammatory cytokines and inhibiting apoptosis through regulation of IL-6/STAT3/NF-κB signaling pathway, which may represent a novel approach to treat CD and provide alternative choice for disorders associated with CD.

## Data Availability

The datasets used during the present study are available from the corresponding author upon reasonable request.

## Abbreviations

CD: Crohn’s disease
RD: Resina Donis
LB: Loureirin B
TNBS: 2,4,6-trinitrobenzenesulfonic acid solution
PVDF: polyvinylidene difluoride membrane
